# Net Portal Appearance of Amino Acids in Iberian and Landrace Pigs Fed Different Protein Content in the Diet

**DOI:** 10.3390/ani13071263

**Published:** 2023-04-05

**Authors:** Manuel Lachica, José Miguel Rodríguez-López, Lucrecia González-Valero, Ignacio Fernández-Fígares

**Affiliations:** 1 Department of Nutrition and Sustainable Animal Production, Estación Experimental del Zaidín, CSIC, San Miguel 101, Armillla, 18100 Granada, Spain; 2 Départment Sciences Agronomiques et Animales, Institut Polytechnique LaSalle Beauvais-Esitpa, 19 Rue Pierre Waguet, BP 30313, 60026 Beauvais, France

**Keywords:** amino acid, net portal appearance, pig, portal-drained viscera

## Abstract

**Simple Summary:**

Iberian pig (*Sus mediterraneus*) is a rustic breed that thrives in the Mediterranean forest in the Southwest of the Iberian Peninsula, traditionally raised in open range, grazing acorn and grass. The information on nutrient requirements of Iberian pigs compared with modern breeds is scarce and derived from modern breeds, even though there are evidences that they have distinct metabolic and nutritional features, showing a much lower growth rate and protein deposition. This study attempts to shed light on this issue. By using net portal appearance, the intestinal absorption of nutrients is considered, and therefore the nutrient availability for pig growth is estimated. The present study focused on the net portal appearance of amino acids in Iberian pigs compared to a modern breed known as Landrace. Iberian pigs showed a lower net portal appearance of amino acids than Landrace pigs, regardless of the content of protein in the diet. Differences in net portal appearance of amino acids may partially explain the lower growth rate of Iberian pigs compared to modern breeds, maybe as result of a greater use of amino acids by the gastrointestinal tract. Strategies supplementing key gut amino acids in support of gut function may improve pig performance during the productive period.

**Abstract:**

Iberian pigs have low rates of muscle protein deposition compared with modern breeds. Differences in net portal appearance (NPA) of amino acids (AA) might partially explain that. NPA of AA was measured in six Iberian and six Landrace gilts (28 kg) fitted with catheters in portal and mesenteric (para-aminohippuric acid infusion) veins, and carotid artery. Blood samples from porta and artery were simultaneously taken at 0, 0.5, 1, 1.5, 2, 2.5, 3, 3.5, 4, 5, and 6-h after feeding two isoenergetic diets (14–14.5 MJ metabolizable energy/kg dry matter) with different crude protein (145 (LCP) and 187 (HCP) g/kg dry matter) content. NPA of essential AA (EAA) and non-essential AA (NEAA) was lower (p < 0.05) in Iberian than Landrace pigs, and in LCP than HCP diet. Fractional absorption (NPA/AA intake) of EAA, NEAA, and total AA was, respectively, 36, 49, and 44% lower in LCP than HCP diet in Iberian pigs; and 8, 2, and 4% greater in Landrace pigs. Fractional absorption of EAA, NEAA, and total AA was 42, 68, and 60% lower in Iberian than Landrace pigs fed LPC diet; and 1, 36, and 26% when fed the HCP diet. NPA of AA may partially explain the low growth rate of Iberian pigs.

## 1. Introduction

Iberian pig (*Sus mediterraneus*) is an autochthonous breed that thrives in the Mediterranean forest in the Southwest of the Iberian Peninsula (Spain and Portugal), consuming acorns (1.3–6 kg dry matter (DM)/day [1]) from oak-trees (*Quercus ilex rotundifolia* and *Quercus suber*) complemented with pasture, and provides products of exceptional organoleptical properties [2]. This particular agroecosystem is denominated *dehesa*. The productive cycle of the Iberian pig is orientated towards a final grazing/fattening period at the *dehesa* named *montanera*. Nowadays, productive cycle follows a semi-extensive system offering balanced compound feed from weaning to about 100 kg body weight (BW) then pigs are further finished outdoors at *montanera* up to 160 kg BW in fall and winter. However, most Iberian pigs are raised under a standard balanced diet during the whole productive period as the stocking rate at *montanera* is very low, limiting the number of pigs under extensive conditions.

The information on nutrient requirements of Iberian pigs compared with modern breeds is scanty, even though there are evidences that they have distinct metabolic [3] and nutritional features [4].

The gastrointestinal tract has a huge metabolic influence relative to its weight; moreover, its relative contribution to whole-body protein synthesis is disproportionately elevated [4]. Comparative studies between Iberian and Landrace pigs [5] reported greater splanchnic tissue weight/BW ratio in Iberian than Landrace pigs although greater portal drained viscera heat production has been reported for the latter [6]. The Iberian pig has a reduced capacity for protein deposition at maximal growth compared with lean breeds [7], with whole-body protein synthesis and degradation rates lower in Iberian than in Landrace pigs when given an optimal dietary amino acid (AA) supply [8]. Surprisingly, muscle fractional protein synthesis was greater in Iberian than in Landrace gilts [5], although muscles were smaller for the former. There is controversial information about the digestive features of Iberian pigs. The post-weaning N digestibility was lower in Iberian compared with Landrace pigs [8] and apparent total tract digestibility of crude protein (CP) did not differ in piglets [9]. Although ileal digestibility of AA was measured in Iberian pigs [10,11], available information on net portal appearance (NPA) is very scarce [12]. Differential capacity of AA absorption and/or use by the gastrointestinal tract would imply diverging amount of nutrients available to the peripheral tissues. Again, very little information is available in the literature relating comparative capacity of AA absorption in native and modern breeds which is a good approach to evaluate bioavailability of AA. Differences in NPA of AA might partially explain the lower growth rate reported in Iberian compared to modern breeds. The main objective of this study was to investigate differences between Iberian and Landrace growing pigs on NPA of AA after feeding diets with different CP content. The information could be used to establish recommendations of AA supplementation for Iberian pigs.

## 2. Materials and Methods

### 2.1. Animals and Diet

Six Iberian (Silvela strain; Sánchez Romero Carvajal, Jabugo S.A., Puerto de Santa María, Spain) and six Landrace (Granja El Arenal, Córdoba, Spain) gilts of similar age and BW (80 ± 3 vs. 75 ± 2 days and 25.3 ± 0.4 vs. 25.6 ± 0.9 kg BW for Iberian and Landrace pigs, respectively) were utilized.

One week before surgery, pigs were housed in individual pens in a controlled environment room (21 ± 1.5 °C) with ad libitum access to an appropriate standard diet (145 g CP/kg DM and 14.3 MJ metabolizable energy (ME)/kg DM for Iberian [7]; and 187 g CP/kg DM and 14.3 MJ ME/kg DM for Landrace [13]) based on barley-soybean meal was offered with free access to water. After surgery, pigs were fed (2.4 × ME for maintenance; 444 kJ/kg^0.75^ BW/day [14]) twice a day at 09:00 (0.25 of the daily ration) and at 15:00 h (0.75 of the daily ration). Composition and chemical analysis of diets is shown in Table 1.

DM (no. 934.01) and total ash (no. 942.05) analysis were performed by standard procedures [15]. Total N was determined according to the Dumas’ method, by total combustion in TruSpec CN equipment (Leco Corporation, St. Joseph, MI, USA) and CP determined as total N × 6.25. Gross energy was measured in an isoperibolic bomb calorimeter (Parr Instrument Co., Moline, IL, USA). AA composition of diets (Alanine (Ala), Aspartic acid (Asp), Cysteine (Cys), Glutamic acid (Glu), Glycine (Gly), Histidine (His), Isoleucine (Iso), Leucine (Leu), Lysine (Lys), Methionine (Met), Phenylalanine (Phe), Proline (Pro), Serine (Ser), Threonine (Thr), Tyrosine (Tyr), and Valine (Val)) was determined after protein hydrolysis in 6 mol/L hydrochloric acid plus 10 g/kg phenol in sealed tubes at 110 °C for 24 h, by high performance liquid chromatography (HPLC) using the Waters Pico-Tag method for hydrolysates which involves pre-column with phenylisothiocyanate [16] and a Waters Nova-Pak C18 phase reverse column (4 µm, 3.9 × 150 mm). Cys and Met in the diet were determined as cysteic acid and Met sulphone, respectively, obtained by oxidation with performic acid before protein hydrolysis [17]. Tryptophan (Trp) was not determined. Free AA in plasma were determined by HPLC using the Waters Pico-Tag method for physiological AA using a reverse phase column (Waters Pico-Tag, 3.9 × 300 mm) and pre-column derivatization with phenylisothiocyanate [16].

### 2.2. Experimental Schedule and Measurements

Pigs were adapted to close contact with the staff involved with sampling to reduce stress to a minimum. Approximately at 29 kg BW pigs were fasted and water removed 24 h before the installation of chronic indwelling catheters in portal and mesenteric veins, and carotid artery. Briefly, pigs were fed 24 h prior to surgery with free access to water. General anaesthesia was induced using an intramuscular (i.m.) combination of Ketamine (15 mg/kg BW) and Azaperone (2 mg/kg BW), and maintained throughout the surgical procedure by administering isoflurane (0.5–2%) and O_2_ (22–44 mL/kg BW/min) through a face mask. A dose of 5 mL (i.m.) of N-butyl hyoscine bromide + sodium metamizol (Buscapina Compositum) was administered as an analgesic and anti-spasm agent. Incision areas were clipped closely, washed and scrubbed three times using iodine soap before the gilt was moved to the surgery room table. Incision area was sprayed with povidone iodine (7.5%) and alcohol (70%). Strict aseptic and sterile conditions were applied throughout the whole procedure. First, the portal vein was catheterized through the visceral side of the liver left lateral lobe; the catheter was inserted 6 cm towards the entry of the portal vein in the liver and sutured to the parenchyma with non-absorbable suture. Second, a branch of mesenteric vein was located, and catheter installed after separating the surrounding connective tissue, a small puncture was made and the catheter inserted 10 cm downstream into the vein. The catheter was secured by two non-absorbable sutures around the vessel. Third, the carotid artery was not tied (occluded) but the catheter was secured in place with a purse-string non-absorbable suture to allow blood flow and avoid later local infections. After patency was confirmed by flushing with physiological saline containing 5 IU heparin/mL, all the catheters (mesenteric vein and carotid artery: Tygon, i.d. 1.02 mm, o.d. 1.78 mm, length 65 cm; portal vein: Tygon, i.d. 1.27 mm, o.d. 2.29 mm, length 65 cm) were filled with physiological saline containing 250 IU heparin/mL and locked. Pigs were moved to metabolic cages. They resumed their normal eating habits (fed at 25, 60, and 100% of their preoperative intake for first, second, and third day after surgery, respectively) usually in three days. Rectal temperature was normal. Amoxicillin was given i.m. for 4 days after surgery. Around ten days later, stiches were removed and pigs were considered fully recovered. Detailed description of the catheters design, construction and maintenance, surgical procedure, and post-surgery care of pigs was published elsewhere [18]. Blood sampling commenced when pigs were completely recovered from surgery at 32 kg average BW, and following the identical feeding schedule described above in a crossover design with a week adaptation for both diets. An initial 15 mL pulse dose of para-aminohippuric acid (PAH; 2% *w/v*; Sigma-Aldrich Química S.A., Madrid, Spain) was administered into mesenteric vein 45 min before blood sampling, followed by a continuous infusion of 0.8 mL/min using a syringe pump (Model 33, Harvard Apparatus Inc., Holliston, MA, USA). Apyrogenic filters (MILLEX GP, Syringe Driven Filters Unit, 0.22 µm; Millipore, Carringtwohill, Ireland) fitted infusion syringes. A blood sample using 4.5 mL heparinized tubes (Monovette VetMed, Sarstedt, Nümbrecht, Germany) was taken simultaneously (for haematocrit, PAH and AA analyses) from carotid artery and portal vein 0, 0.5, 1, 1.5, 2, 2.5, 3, 3.5, 4, 5, and 6 h after feeding 0.25 of total daily ration. Haematocrit was determined using a microcentrifuge (11,500× *g* for 5 min; Biocen, Orto-Alresa, Ajalvir, Madrid, Spain). Plasma was obtained by centrifugation (4 °C and 1820 × *g* for 30 min; centrifuge 5810R Eppendorf, Hamburg, Germany) and stored at −20 °C until PAH [19] and AA analyses. Thr was not quantified in plasma because of co-elution with ammonia peak.

The portal blood flow (PBF) and portal plasma flow (PPF) were determined by the indicator dilution method using haematocrit and plasma PAH concentrations [20]. The PBF and NPA of AA were calculated according to the Fick principle of arterio-venous concentration difference and flow rate [21]. The NPA of AA was calculated by multiplying the porto-arterial plasma concentration difference of AA by PPF. Fractional absorption for each AA was calculated as the ratio NPA/AA intake during the 6 h postprandial.

### 2.3. Statistical Analyses

Data were subjected to ANOVA using the MIXED procedure of SAS (SAS Inst. Inc., Cary, NC, USA). The statistical model applied included the fixed effect of diet, breed, sampling time, and corresponding interactions. Time of sampling within the pig was considered as a repeated measure. Concentration at time zero of the AA was included as a covariate in the statistical analysis. The differences were considered significant when *p* < 0.05.

## 3. Results

PPF was lower (*p* < 0.001) in Iberian than Landrace (539 and 859 mL/min, respectively) pigs; and greater (*p* < 0.001) in diet HCP than LCP (759 and 639 mL/min, respectively). In addition, PPF was influenced by time (*p* < 0.001). The maximum PPF peak for Iberian and Landrace was 0.5 and 1 h after diet ingestion, respectively, reaching a maximum value of 668 for Iberian and 996 mL/min for Landrace gilts; with respect to the CP content, PPF reached the maximum at 0.5 and 1 h for HCP (892 mL/min) and LCP (739 mL/min) diets, respectively. After reaching the peak, it decreased thereafter until basal rate. No difference in the time needed to eat 0.25 of daily ration was found between breeds.

NPA of AA during the 6 h sampling is shown in Table 2.

**Figure 1:**
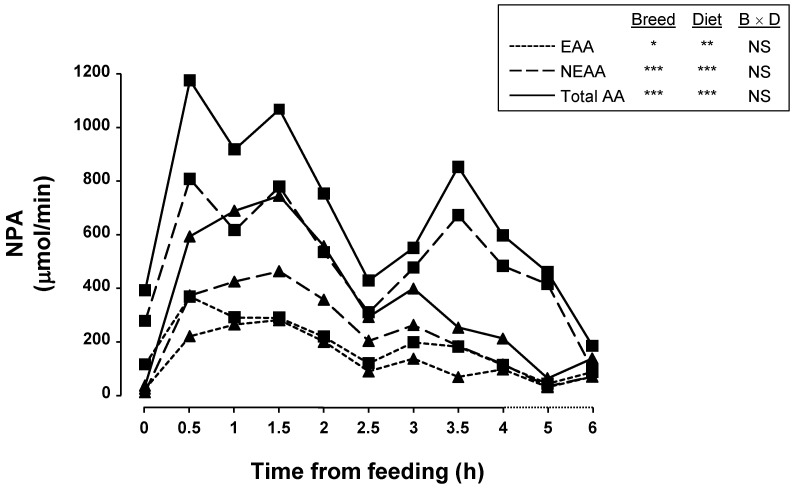
NPA of EAA, NEAA and total AA along a 6 h sampling in Iberian (▲) and Landrace (■) pigs (*n* = 6/breed (B)) fed two diets (D) of different protein content. * *p* < 0.05, ** *p* < 0.01, *** *p* < 0.001, NS = not significant (*p* > 0.05).

There were no breed × diet interactions for essential AA (EAA), non-essential AA (NEAA), and total AA. The overall NPA of the EAA His, Leu, Phe and Trp, and NEAA Ala, Asparaguine (Asn), Cys, Glu, Gly, Hydroxiproline (Hyp), Pro, Ser, Taurine (Tau), and Tyr was lower (*p* < 0.05) and that of Ornithine (Orn) was greater for Iberian than Landrace pigs during the 6-h postprandial period. When pigs consumed a HCP diet the overall NPA of the EAA Arg, His, Leu, Lys, Met, Phe, Trp, and Val, and NEAA Ala, Asn, Gly, Ser, Tau, and Tyr was greater (*p* < 0.05) and tended to be greater for Glu (*p* = 0.07). NPA was negative for Glutamine (Gln) and Glu. NPA of EAA and NEAA was lower (21 and 47%, respectively; *p* < 0.05) for Iberian than Landrace pigs as well as for LCP compared with HCP diets (34 and 36%, respectively; *p* < 0.01). NPA of NEAA quantitatively represented the major part of NPA of total AA (65 and 74% for Iberian and Landrace pigs, respectively). NPA of EAA, NEAA, and total AA is shown in Figure 1. Overall, NPA rose until 1.5 h after feeding, decreased until 2.5 h and increased again until 3.5 h to achieve a second peak decreasing thereafter to a level similar to preprandial.

Fractional absorption (NPA/AA intake) is displayed in Table 3. Negative fractional absorption was observed for Glu, and in the case of Cys for Iberian pigs fed a LCP diet. In Iberian pigs, fractional absorption was 36, 49, and 44% lower for EAA, NEAA, and total AA, respectively; whereas in Landrace pigs it was 8, 2, and 4%, respectively, greater in the LCP than HCP diet. When LCP was fed, fractional absorption was 42, 68, and 60% lower in Iberian than Landrace pigs. On the other hand, when the HCP diet was fed, fractional absorption was 1, 36, and 26% lower in Iberian than Landrace pigs for EAA, NEAA, and total AA, respectively.

## 4. Discussion

In the present study, the animals were fed 0.25 of the daily ration, proportional to the measurement period (6 h). Overall, NPA of AA in the present study reached a maximum during the first quarter of the sampling length in agreement with data reported in the literature [12,22,23,24]. Compared with a previous study made in our lab [12], time to reach the peak was longer as a consequence of the greater amount of feed offered in the present study as there is an inverse relationship between amount of nutrients intake and the necessary time to be absorbed by the portal-drained viscera (PDV). PPF (or PBF) measured in our conditions was within the range of measurements in Landrace and Iberian pigs fed an amount of feed proportional to the measurement period [6,12,25]. Nevertheless, PBF depends upon the experimental conditions. Thus, PBF was affected by the amount of feed intake [26] or by the adaptation to a low CP content [12]. Indeed, a relation between feed intake and blood flow has been proved [27,28]. The decreased PPF (*p* < 0.001) in Iberian vs. Landrace pigs may indicate differences in PDV physiology associated with breed, as supported by the low energy efficiency for protein deposition [7] and greater protein turnover in Iberian compared with Landrace pigs.

It is difficult to compare results of NPA of AA from different studies as NPA is affected by numerous factors (protein content, fiber, ME intake, etc. [29]). Indeed, greater NPA of AA were reported in Large White pigs fed diets of similar CP content (120–240 g/kg DM [30]) than in the present study and lower values NPA of AA were found in Iberian pigs fed diets with reduced CP content (52 g/kg DM [12]).

It is interesting to point out that NPA and fractional absorption of Lys in our study were not low compared to other EAA. Although according to chemical score Lys was the first limiting AA in milk-protein fed to piglets [31], the limiting EAA for extra-intestinal protein deposition were Thr and Met according to net portal AA balance [32]. Amongst EAA, NPA of AA was similar (*p* > 0.05) for Lys and Met between breeds, the lower values corresponded to Trp followed by His, Phe and Leu, and were always greater (*p* < 0.05) in Landrace pigs. Trp could be considered the first limiting AA followed by Met. Dietary Arg is highly metabolized during intestinal transport to provide Orn and Citrulline (Cit) in pigs [33,34], but unlike Gln, Arg is not oxidized at the small intestine [35]. We have no explanation for the greater (*p* < 0.05) NPA of Orn in Iberian than in Landrace pigs since Arg was not different (*p* > 0.1) as well as the NPA of Cit. Reverter et al. [36] reported that in pigs Orn, Cit, Hyp, and Tau appeared in the hepatic portal blood without being present in the diets, which could be due to metabolism in the arteries and to modification of absorbed AA in the digestive tract changing the proportions of absorbed AA relative to the dietary content. Cit and Orn are metabolites of the urea cycle synthesized at the intestinal wall in large amounts [37]. Fractional absorption and NPA of AA was always positive except for Gln and Glu. Fractional absorption for Glu was negative, that is, the net use of these AA by the PDV was greater than the dietary intake, implying a high rate of metabolism in gastrointestinal tissues. Certainly, a greater rate of Glu metabolism was observed in Iberian than in Landrace pigs and with pigs fed the LCP diet compared to the HCP diet. Analogously, negative fractional absorption of Glu was reported when Iberian pigs were fed with a diet of very low CP content (52 g/kg DM [12]). Fractional absorptions between 0.5–1 for most EAA have been reported using diets of similar protein content [36,38,39].

Glu, Gln and, to a lesser extent, Asp appear to be significant oxidative fuels in the intestine, as reflected by their low or even negative NPA. Despite the importance of glucose as oxidative fuel, Stoll et al. [40] obtained that the proportion of glucose oxidized completely to CO_2_ was substantially less than that of either Gln or Glu in piglets, in agreement with the positive NPA of glucose reported by Fernández-Fígares et al. [41] in Iberian pigs fed acorn. A nearly complete first-pass removal of dietary Glu and Asp has been reported in crossbred [32,39] or Iberian [12] pigs; it was also reported negative NPA of Glu + Gln but not of Asp [36].

On the other hand, NPA of Ala, Gly, and Pro were the largest in the conditions of our experiments; with values, respectively, 42, 49, and 39% lower in Iberian than in Landrace pigs and their fractional absorption was always above 1 (that is, there was a net synthesis at the PDV level) except for Gly (0.832) and Pro (0.734) in Iberian pigs fed the LCP diet. Similarly, Hu et al. [39] reported fractional absorption exceeding 1 of Ala and Gly, pointing out that these values are only possible if other AA are transaminated in the intestinal mucosa during the absorptive process. High NPA of Ala and Gly are the result of metabolic processes in the gut wall [22,32]. Gln and Glu act as precursors for Pro synthesis [42]. In post-absorptive state, the intestine releases large amounts of Cit together with Ala and Pro in pigs [43] indicating de novo synthesis by the gut. However, NPA of Cit and its endogenous precursor Arg were not low in our study. In a previous study in Iberian pigs fed a very low CP diet [12], the low NPA of Cit was paralleled by a negative NPA of Arg. Indeed, dietary Cit supplementation was more efficient to increase Arg availability than Arg supplementation in mice [44]. Orn originates from the metabolism of dietary and blood Arg [45], in agreement with the large Orn concentration in portal blood and positive NPA in this and other studies [29]. The absorption of dietary Cys into portal blood is very limited in young pigs (less than 0.20 of dietary intake), implying extensive intestinal use of Cys in first-pass [32,46], as a precursor for glutathione synthesis. In all the cases, NPA of AA was both, numerically or statistically (*p* < 0.05) lower in Iberian compared to Landrace pigs; on the contrary, NPA of Orn in Iberian was 38% greater than Landrace pigs.

Overall, we found that AA were used by PDV to a greater extent in Iberian than Landrace pigs as indicated by their lower fractional absorption, maybe as a result of the greater protein turnover showed by Iberian pigs together with the heavier relative weights (as a proportion of BW) of stomach, large intestine, and total gastrointestinal tract of Iberian compared to Landrace gilts [5]. Iberian pigs showed a lower fractional absorption for EAA, NEAA, and total AA for the LCP diet, when compared to the HPC diet, which is in agreement with the greater gut endogenous protein secretion and AA reabsorption after digestion expected in pigs fed diets with increasing protein content [47]. It seems that Iberian pigs AA utilization by PDV is increased when diet protein content is low, maybe as an adaptation to the protein shortage in their natural environment (Mediterranean woodland -dehesa-) during the grazing period (montanera). Fractional absorption was comparable to literature values (0.67 [12], 0.64 [32], or 0.69 [33]). As previously mentioned, except for Orn, NPA of AA (EAA, NEAA and total AA) was lower (*p* < 0.05) in Iberian than in Landrace pigs indicating an overall greater catabolism of AA so that less AA are available for productive tissues. Reverter et al. [36] reported that some AA such as Cys seemed to be absorbed to a lesser extent on certain diets. This discrepancy between digested and absorbed AA could be due to synthesis of protein from AA in the intestinal wall and/or catabolism of absorbed AA by the intestinal mucosa and the rest of the PDV, transport via blood cells [48], or absorption of oligopeptides. There appears to be no available quantitative data on the absorption of small peptides in pigs, and data from ruminants are conflicting. Some authors have pointed out this possibility, indicating that transport of peptides may be an important mechanism for inter-organ metabolism of some AA [33]. Nevertheless, Fernández-Fígares et al. [49] reported that a major proportion of AA appearing in portal blood of growing Iberian pigs was in the form of peptide AA, small peptides representing on average 72% of total AA appearing in the portal vein.

In a previous study, Lachica et al. [12] established a correspondence between the low NPA of AA and fractional absorption, and the poor CP content of diet. A nutrient deficient diet may to reduce intestinal villi height of pigs [50], which may have further implication in Iberian pigs, that have lower ileal villi length and villi-to-crypt ratio compared to Landrace × Large White pigs [51]. This fact could explain the lower NPA of AA in Iberian compared to Landrace pigs.

Lys was the first limiting AA in milk-protein fed to piglets according to chemical score [31] while Thr and Met were the limiting AA according to net portal AA balance [32]. Amongst EAA, Trp showed the lowest NPA (*p* < 0.05) while Trp and Val had the lowest fractional absorption particularly in Iberian pigs (0.418) so both AA could be considered the first limiting AA (Trp for Iberian and Landrace pigs, and Val for Iberian pig) in the current study. Comparative studies carried out in Iberian and Landrace pigs [5] showed that splanchnic tissues represented 10.2 and 8.3% of total BW, respectively, and their contribution to whole-body protein synthesis was, respectively, 48 and 32%. They found no differences in protein synthesis rates of visceral tissues between both breeds although protein synthesis rate of liver and duodenum were 6 to 10 times greater than those of muscle for both genotypes. It could be speculated that the lower PBF in Iberian may partially be responsible of the lower growth rate relative to Landrace pigs, especially taking into account the greater splanchnic tissues mass relative to BW in Iberian pigs. This would be in accordance with the low energy efficiency for protein deposition reported for Iberian pigs [7]. In Iberian pig the maximum capacity for protein deposition is far less than values found in lean and conventional genotypes irrespective of BW range, consequently, protein deposition is a very inefficient process. The low energetic efficiency of protein deposition might be attributed partly to the comparatively greater muscle protein turnover rate in Iberian pigs [5]. Results support the existence of genetic variation in the efficiency of energy use but the ultimate causes of low ME utilization for protein deposition (k_p_) values observed in the Iberian pig remain unexplained [4].

The biological explanation for the differences in the proportion of dietary AA utilized by the PDV is only partially understood and requires further studies to specifically establish the functional purpose of each AA.

## 5. Conclusions

Overall, the net portal appearance of amino acids of Iberian pigs was lower than Landrace pigs independently of the content of protein in the diet. The content of protein in the diet increased the net portal appearance of amino acids. Strategies to supplement key gut-amino acids in support of gut function may improve pig performance, so it could be recommended during the productive period in Iberian pigs.

## Figures and Tables

**Table 1:** Composition (g/kg) and chemical analysis (dry matter (DM) basis) of low (LCP) and high crude protein (HCP) content diets.

	LCP	HCP
Barley grain	866	802
Soybean meal	96.2	125
Fish meal	4	40
Calcium carbonate	8	8
Calcium acid phosphate	12	12
Sodium chloride	5	5
L-Lys	4.32	3.92
Thr	1.05	0.96
Met	0.47	0.21
Vitamin-mineral premix ^1^	3	3
Chemical analysis		
DM, g/kg	870.12	875.14
Ash, g/kg	58.41	59.96
CP, g/kg	145.43	187.12
Gross energy, MJ/kg	17.83	18.08
Amino acids		
EAA ^2^	58.0	73.7
Arg	9.86	13.3
His	3.93	5.01
Ile	4.38	5.74
Leu	8.31	11.0
Lys	11.5	13.6
Met	2.51	3.47
Phe	5.52	7.04
Thr	6.51	7.50
Val	5.52	7.10
NEAA ^2^	60.1	77.2
Ala	4.95	7.12
Asp	7.78	11.4
Cys	2.01	2.51
Glu	21.9	26.6
Gly	5.03	7.00
Pro	9.13	10.7
Ser	5.00	6.55
Tyr	4.27	5.22
Total AA ^2^	118.1	150.8

^1^ Provided per kg of complete diet: 9836 international units (IU) vitamin A as retinyl acetate; 2253 IU vitamin D_3_ as cholecalciferol; 2.52 IU vitamin E as DL-𝛼-tocopheryl acetate; 1.5 mg menadione sodium bisulfite; 0.15 mg thiamine; 3 mg riboflavin; 0.15 mg pyridoxine; 15 μg cyanocobalamin; 15 μg folic acid; 22.5 mg nicotinic acid; 15 mg D-pantothenic acid as calcium pantothenate; 15 mg Mn as MnSO_4_ × 4H_2_O; 75 mg Fe as FeSO_4_ × 7H_2_O; 120 mg Zn as ZnO; 450 μg I as KI; 60 mg Cu as CuSO_4_ × 5H_2_O; and 300 μg Co as CoSO_4_ × 7H_2_O). ^2^ Sum of essential (EAA: Arginine (Arg), Histidine (His), Isoleucine (Iso), Leucine (Leu), Lysine (Lys), Methionine (Met), Phenylalanine (Phe), Threonine (Thr), and Valine (Val)), non-essential (NEAA: Alanine (Ala), Aspartic acid (Asp), Cysteine (Cys), Glutamic acid (Glu), Glycine (Gly), Proline (Pro), Serine (Ser), and Tyrosine (Tyr)), and total amino acids (AA).

**Table 2:** Net portal appearance (NPA; µmol/min) of EAA, NEAA and total AA in Iberian and Landrace pigs receiving either LCP (145 g CP/kg DM) or HCP (187 g CP/kg DM) diets.

	Breed		Diet			*p*-Value		
	Iberian	Landrace	LCP	HCP	SEM ^1^	Breed	Diet	Breed × Diet
EAA	146.8	186.3	132.5	200.8	10.71	0.0388	0.0015	0.1244
Arg	25.7	30.4	20.2	35.9	3.03	0.4469	0.0119	0.5154
His	9.06	11.8	8.82	12.1	0.584	0.0203	0.0067	0.3054
Ile	13.6	12.0	12.3	13.3	1.03	0.4525	0.6259	<0.0001
Leu	23.7	37.4	25.1	36.0	1.60	<0.0001	0.0009	0.1877
Lys	36.1	40.5	29.6	46.9	2.48	0.3850	0.0008	0.3674
Met	7.53	7.96	6.55	8.94	0.449	0.6419	0.0094	0.0001
Phe	15.3	21.5	16.0	20.9	0.82	0.0003	0.0039	0.0401
Trp ^2^	1.65	5.07	2.03	4.69	0.491	0.0008	0.0084	0.8918
Val	14.2	19.7	11.9	22.1	2.14	0.2050	0.0200	0.6487
NEAA	275.3	519.3	309.9	484.7	24.37	<0.0001	0.0009	0.2948
Ala	93.9	161.1	105.1	149.9	5.88	<0.0001	0.0002	0.5512
Asn ^2^	43.1	65.3	47.0	61.3	2.72	0.0001	0.0100	0.4796
Asp	5.60	7.40	5.79	7.20	0.606	0.1452	0.2528	0.4883
Cit ^2^	13.7	11.0	12.9	11.9	0.90	0.1343	0.5803	0.2916
Cys	3.13	15.1	8.92	9.34	1.973	0.0031	0.9178	0.0289
Gln ^2^	−18.6	−20.1	−22.4	−16.3	0.43	0.8666	0.5078	0.8226
Glu	−29.2	−7.29	−26.3	−10.2	4.284	0.0149	0.0738	0.8166
Gly	73.8	144.0	80.4	137.4	6.25	<0.0001	<0.0001	0.0695
Hyp ^2^	1.47	6.83	3.55	4.75	0.461	<0.0001	0.2011	0.0299
Orn ^2^	11.6	7.21	8.15	10.7	0.908	0.0180	0.1706	0.3447
Pro	46.9	77.4	55.9	68.3	3.72	0.0001	0.1015	0.1528
Ser	18.2	30.6	18.7	30.1	2.00	0.0026	0.0055	0.1236
Tau ^2^	1.79	5.63	1.84	5.58	0.651	0.0041	0.0052	0.6649
Tyr	9.87	15.1	10.3	14.7	0.739	0.0006	0.0042	0.6566
TAA	422.1	705.6	442.4	685.5	32.21	<0.0001	0.0008	0.1326

^1^ Standard error of mean; *n* = 6 pigs per breed. ^2^ Tryptophan (Trp), Asparagine (Asn), Citrulline (Cit), Glutamine (Gln), Hydroxiproline (Hyp), Ornithine (Orn), Taurine (Tau).

**Table 3:** Fractional absorption (NPA/AA intake; mmol) of EAA, NEAA and total AA in Iberian (Ib; *n* = 6) and Landrace (Ld; *n* = 6) pigs receiving either LCP (145 g CP/kg DM) or HCP (187 g CP/kg DM) diets.

	AA Intake		Fractional Absorption					
	LCP	HCP	Ib-LCP	Ib-HCP	Ld-LCP	Ld-HCP	SEM	*p*-Value
EAA ^1^	76.7	99.4	0.449 ^a^	0.705 ^b^	0.774 ^b^	0.715 ^b^	0.0426	0.0108
Arg	12.3	16.7	0.461	0.764	0.716	0.779	0.0842	0.3553
His	5.52	7.07	0.445 ^a^	0.575 ^ab^	0.705 ^b^	0.652 ^b^	0.0338	0.0283
Ile	7.27	9.59	0.400 ^a^	0.717 ^b^	0.817 ^b^	0.282 ^a^	0.0465	<0.0001
Leu	13.8	18.4	0.420 ^a^	0.613 ^ab^	0.890 ^c^	0.798 ^bc^	0.0357	<0.0001
Lys	17.1	20.4	0.529 ^a^	0.830 ^b^	0.716 ^ab^	0.827 ^b^	0.0472	0.0778
Met	3.66	5.10	0.440 ^a^	0.749 ^b^	0.847 ^b^	0.515 ^a^	0.0375	0.0002
Phe	7.28	9.33	0.549 ^a^	0.752 ^b^	1.029 ^c^	0.856 ^bc^	0.0349	<0.0001
Val	10.3	13.3	0.286	0.550	0.547	0.645	0.0678	0.2694
NEAA ^1^	108.3	139.7	0.419 ^a^	0.819 ^b^	1.301 ^c^	1.277 ^c^	0.0477	<0.0001
Ala	12.1	17.5	2.232 ^a^	2.318 ^a^	4.020 ^b^	3.848 ^b^	0.1385	<0.0001
Asp	12.7	18.8	0.151	0.113	0.177	0.164	0.0147	0.4503
Cys	3.61	4.54	−0.147 ^a^	0.615 ^a^	1.923 ^b^	0.865 ^ab^	0.1812	0.0006
Glu	32.4	39.6	−0.425 ^a^	−0.184 ^b^	−0.159 ^b^	−0.003 ^b^	0.0451	0.0460
Gly	14.6	20.4	0.832 ^a^	2.006 ^b^	3.136 ^c^	2.838 ^bc^	0.1372	<0.0001
Pro	17.3	20.4	0.734 ^a^	1.035 ^ab^	1.597 ^c^	1.383 ^bc^	0.0712	0.0001
Ser	10.4	13.6	0.324 ^a^	0.712 ^b^	0.973 ^b^	0.873 ^b^	0.0619	0.0008
Tyr	5.13	6.31	0.516 ^a^	0.707 ^ab^	0.933 ^b^	0.970 ^b^	0.0482	0.0033
Total AA ^1^	182.0	235.2	0.439 ^a^	0.784 ^b^	1.100 ^c^	1.060 ^c^	0.0784	<0.0001

^1^ Sum stablished only for analyzed dietary AA. ^a,b,c^ Values within a row with unlike superscript letter were significantly different.

## Data Availability

The data presented in this study are available on request from the corresponding author.
